# Human endogenous retrovirus K in the respiratory tract is associated with COVID-19 physiopathology

**DOI:** 10.1186/s40168-022-01260-9

**Published:** 2022-04-22

**Authors:** Jairo R. Temerozo, Natalia Fintelman-Rodrigues, Monique Cristina dos Santos, Eugenio D. Hottz, Carolina Q. Sacramento, Aline de Paula Dias da Silva, Samuel Coelho Mandacaru, Emilly Caroline dos Santos Moraes, Monique R. O. Trugilho, João S. M. Gesto, Marcelo Alves Ferreira, Felipe Betoni Saraiva, Lohanna Palhinha, Remy Martins-Gonçalves, Isaclaudia Gomes Azevedo-Quintanilha, Juliana L. Abrantes, Cássia Righy, Pedro Kurtz, Hui Jiang, Hongdong Tan, Carlos Morel, Dumith Chequer Bou-Habib, Fernando A. Bozza, Patrícia T. Bozza, Thiago Moreno L. Souza

**Affiliations:** 1 Laboratory on Thymus Research, Oswaldo Cruz Institute (IOC), Oswaldo Cruz Foundation (Fiocruz), Rio de Janeiro, RJ Brazil; 2 National Institute for Science and Technology on Neuroimmunomodulation (INCT/NIM), Oswaldo Cruz Institute (IOC), Oswaldo Cruz Foundation (Fiocruz), Rio de Janeiro, RJ Brazil; 3 Laboratory of Immunopharmacology, Oswaldo Cruz Institute (IOC), Oswaldo Cruz Foundation (Fiocruz), Rio de Janeiro, RJ Brazil; 4 Center for Technological Development in Health (CDTS), National Institute for Science and Technology on Innovation on Disease Of Neglected Poppulations (INCT/IDPN), Oswaldo Cruz Foundation (Fiocruz), Rio de Janeiro, RJ Brazil; 5 Laboratory of Immunothrombosis, Department of Biochemistry, Federal University of Juiz de Fora (UFJF), Juiz de Fora, Minas Gerais Brazil; 6 Laboratory of Toxinology, Oswaldo Cruz Institute (IOC), Oswaldo Cruz Foundation (Fiocruz), Rio de Janeiro, RJ Brazil; 7 Instituto de Tecnologia em Imunobiológicos (Bio-Manguinhos), Oswaldo Cruz Foundation (Fiocruz), Rio de Janeiro, RJ Brazil; 8 Instituto de Ciências Biomédicas, Federal University of Rio de Janeiro (UFRJ), Rio de Janeiro, RJ Brazil; 9 Paulo Niemeyer State Brain Institute (IECPN), Rio de Janeiro, RJ Brazil; 10 Evandro Chagas National Institute of Infectious Diseases, Oswaldo Cruz Foundation (Fiocruz), Rio de Janeiro, RJ Brazil; 11 D’Or Institute for Research and Education, Rio de Janeiro, RJ Brazil; 12 MGI Tech Co. Ltd, Building No.11, Beishan Industrial Zone, Yantian District, Shenzhen, 518083 China

## Abstract

**Background:**

Critically ill 2019 coronavirus disease (COVID-19) patients under invasive mechanical ventilation (IMV) are 10 to 40 times more likely to die than the general population. Although progression from mild to severe COVID-19 has been associated with hypoxia, uncontrolled inflammation, and coagulopathy, the mechanisms involved in the progression to severity are poorly understood.

**Methods:**

The virome of tracheal aspirates (TA) from 25 COVID-19 patients under IMV was assessed through unbiased RNA sequencing (RNA-seq), and correlation analyses were conducted using available clinical data. Unbiased sequences from nasopharyngeal swabs (NS) from mild cases and TA from non-COVID patients were included in our study for further comparisons.

**Results:**

We found higher levels and differential expression of human endogenous retrovirus K (HERV-K) genes in TA from critically ill and deceased patients when comparing nasopharyngeal swabs from mild cases to TA from non-COVID patients. In critically ill patients, higher HERV-K levels were associated with early mortality (within 14 days of diagnosis) in the intensive care unit. Increased HERV-K expression in deceased patients was associated with IL-17-related inflammation, monocyte activation, and an increased consumption of clotting/fibrinolysis factors. Moreover, increased HERV-K expression was detected in human primary monocytes from healthy donors after experimental SARS-CoV-2 infection in vitro.

**Conclusion:**

Our data implicate the levels of HERV-K transcripts in the physiopathology of COVID-19 in the respiratory tract of patients under invasive mechanical ventilation.

**Video abstract**

**Supplementary Information:**

The online version contains supplementary material available at 10.1186/s40168-022-01260-9.

## Introduction

Severe acute respiratory coronavirus 2 (SARS-CoV-2), the etiological agent of 2019 coronavirus disease (COVID-19), continuously circulates and has caused over 200,000 deaths per month since its original emergence into the human population [1]. Based on official laboratory-confirmed reports, the case fatality ratio of COVID-19 ranges from 1.5 to 10% in developed and developing countries, respectively, before vaccination [1]. In contrast to other highly pathogenic coronaviruses from the twenty-first century, such as SARS-CoV and Middle East respiratory coronavirus (MERS-CoV), SARS-CoV-2 shedding occurs from the pre-symptomatic period to a few weeks after symptom onset [2]. Longer viral replication favors tissue damage, as shown by the positive correlation between high lactate dehydrogenase (LDH) activity, a marker of cell death, and COVID-19 progression [3]. While type II pneumocytes are targeted and destroyed by the infection and the respiratory parenchyma is harmed, innate and adaptive immunological responses are not always able to prevent further progression to poor clinical outcomes and may even worsen the tissue lesions [4, 5].

During the inflammatory response to human pathogenic coronaviruses, circulating neutrophils and monocytes migrate and infiltrate the lungs [6, 7] and other organs, contributing to potentiating and perpetuating inflammation and eventually exacerbating tissue damage [8–10]. In fact, severe COVID-19 has been associated with increased and uncontrolled release of pro-inflammatory mediators (cytokine storm) so that the resolutive mechanisms are overcome by marked upregulation of IL-6, TNF-alpha, and IL-1-beta [4]. It was reported that MERS-CoV- and SARS-CoV-infected macrophages produce high levels of pro-inflammatory cytokines and chemokines [11, 12], and, more recently, that lung monocytes from patients with severe pneumonia caused by SARS-CoV-2 are potent producers of TNF-alpha and IL-6 [13]. In addition, immune cells that orchestrate the innate and adaptive response, such as monocytes and neutrophils, undergo pyroptosis and NETosis during COVID-19 [14–16]. Consistently, leukopenia and uncontrolled coagulopathy, marked by platelet activation and high D-dimer levels, correlate with COVID-19 severity [17–20]. Several markers of activation are high in monocytes from COVID-19 patients [21, 22], parallel to the diminished expression of HLA-DR, a marker of immune suppression, thus implying that they are involved in the uncontrolled inflammation characteristic of severe COVID-19 [23–25]. Additionally, monocyte chemoattraction seems to play a key role in critical COVID-19, as therapeutic disruption of the chemotactic loop seems to promote clinical benefit [26].

Altogether, SARS-CoV-2-triggered inflammation and hypercoagulability have rapidly been defined as the main features of the natural history of disease progression from mild to severe COVID-19 clinical presentations [17, 18, 27].

To date, the factors described above have been associated with disease progression from mild to severe, but they are limited in explaining the mortality of critically ill COVID-19 patients. Therefore, further investigation is necessary to search for overlooked factors associated with high COVID-19 mortality rates. Although COVID-19 patients who stay in the ICU for weeks are more likely to develop nosocomial infections, mortality is high even for patients who are negative for bacterial infections [28, 29]. Despite the best clinical practice to routinely surveil bacterial infections in the ICU, unculturable and unbiased diagnosed viruses are neglected in daily practice. Thus, the systematic analysis of the virome from critically ill COVID-19 patients is necessary, especially in samples from the lower respiratory tract, where the diverse milieu of microorganisms has not been completely cataloged and is associated with disease physiopathology. Evidence emerging from the virome points to the induction of endogenous retroelements in SARS-CoV-2 infection and their implication in the severity of COVID-19, as Alu retrotransposons, LINE-1 elements, HERV-K, -H, -W, and -FRD were identified either by experimental in vitro infection or from ex vivo samples from patients [30–36]. Thus, we analyzed a cohort of critically ill COVID-19 patients under IMV with sustained SARS-CoV-2 loads, inflammation, and coagulopathy to determine whether their lower respiratory tract virome, beyond coronavirus, could improve the rationalization of patients’ progression. In our study, we identified active expression of HERV-K in the lower respiratory tract and plasma of severe COVID-19 patients. HERV-K levels were higher in patients who died soon after the onset of illness. Increased HERV-K expression in deceased patients was associated with severity markers of COVID-19 physiopathology. By experimental infection in human primary monocytes, SARS-CoV-2 induced HERV-K expression, which was diminished by antivirals against COVID-19 and anti-inflammatory drugs. Our data implicate HERV-K in the physiopathology of critically ill COVID-19 patients.

## Methods

### RNA extraction and RT-qPCR

RNA from TA and plasma was extracted using QIAamp Viral RNA (Qiagen, Germany). Quantitative RT-PCR was performed using GoTaq Probe qPCR and RT-qPCR Systems (Promega, USA) in a StepOne Real-Time PCR System (Thermo Fisher Scientific, CA, USA). The primers, probes, and cycling conditions used to detect SARS-CoV-2 RNA have been described elsewhere [37], with a standard curve for the SARS-CoV-2 N gene (Microbiologics, MN, USA).

For HERV-K analysis, extraction and amplification were performed as described elsewhere [38]. Of note, the RNA concentration was determined (NanoDrop 2000, ThermoFisher Scientific, CA, USA) and adjusted to 10 μg before cDNA synthesis [0.5 μl of oligo (dT)20, 0.5 μl of random hexamer primers, 10 mM dNTPs, First-Strand Buffer, 0.1 M DTT, and 200 U SuperScript III First-Strand Synthesis System (Invitrogen, ThermoFisher Scientific, CA, USA)]. A total of 100 ng of cDNA (NanoDrop 2000, Thermo Fisher Scientific) was used to run 50-cycle real-time PCR [PowerUp SYBR Green Master Mix (Applied Biosystems, Thermo Fisher Scientific) in a StepOne Real-Time PCR System (Thermo Fisher Scientific, CA, USA)].

### Enrichment-dependent SARS-CoV-2 sequencing

Total viral RNA from TA was extracted and quantified with the QIAamp Viral RNA (Qiagen, Germany) and the Qubit RNA BR Assay Kit (Thermo Fisher Scientific, CA, USA), respectively. cDNA libraries were constructed with the ATOPLex SARS-CoV-2 full-length genome panel v1.0 (kindly donated by MGI Tech Co., Shenzhen, China), an amplicon-based strategy to improve sequencing readout. Dual-indexed, single-stranded library pools were converted to DNA nanoballs by rolling circle amplification and submitted to pair-end sequencing (100 nt) on the MGISEQ-2000 platform (recently named DNBSEQ-G400, MGI Tech Co. Ltd., Shenzhen, China).

Genomic sequences were quality scored, filtered, trimmed, and assembled into contigs using Genome Detective (https://www.genomedetective.com/) [39]. Consensus fasta sequences were aligned with ClustalW in Unipro UGENE [40] (version 38), and phylogenies were constructed with Nextclade [41] to assign the emerging clades (Supplementary Table 2).

### Unbiased RNA-seq

For an unbiased RNA-seq, metatranscriptomics approach, total viral RNA samples were applied to the MGIEasy RNA Library Prep Set (MGI Tech Co. Ltd., Shenzhen, China). In brief, RNA was initially fragmented by size (250 bp), reverse-transcribed to DNA, and added to a second strand. Subsequent steps included end repair, adaptor ligation, PCR amplification (to augment the overall library yield), denaturation, and circularization of single-stranded libraries. Pooled libraries were then converted to DNA nanoballs by rolling circle amplification and pair-end sequenced (150 nt) on the MGISEQ-2000 platform (MGI Tech Co. Ltd., Shenzhen, China).

Fastq file processing and virome composition were determined [39], and de novo assembled contigs were compared with reference virus databases (NCBI RefSeq) to obtain similarity indices and assign the species ID. Consensus fasta sequences were generated with the built-in default algorithm (i.e., most frequent base for each alignment position) in Unipro UGENE [40] (version 38) using BAM files.

HERV-K sequences from polymerase, gag, and env were compared with representative genomes deposited in GenBank, and three evolutionary analyses were conducted in MEGA X [42] with a total of 1000 bootstraps (Supplementary Fig. 3). The models for evolutionary analyses were selected upon model-fitting simulation. Models with Bayesian information criterion (BIC) scores were considered to describe the substitution pattern the best. For each model, the corrected Akaike information criterion (AICc) value, maximum likelihood value (InL), and number of parameters (including branch lengths) were obtained. The evolutionary history of HERV-K Gag, Pol, and Env was inferred using the maximum likelihood method and the Tamura-Nei model, the general time-reversible model, and the Hasegawa-Kishino-Yano model all using a discrete gamma distribution.

### Proteomic sample preparation

Tracheal aspirated samples (14 samples, 50 μg each) were lysed in 8 M urea solubilized in 20 mM ammonium bicarbonate pH 7.9 containing a complete mixture of protease and phosphatase inhibitors (Roche, Switzerland). After centrifugation at 14,000 RCF for 20 min, the supernatants were transferred to new tubes and heated at 32 °C for 30 min under 600-rpm agitation. Proteins were reduced with 5 mM dithiothreitol for 60 min at 32 °C and alkylated in 14 mM iodoacetamide for 40 min at room temperature in the dark. Samples were then diluted to 1 M urea, and 1 μg of modified trypsin (Promega, WI, EUA) (1:50 w/w — trypsin:substrate ratio) was added. Each sample was then incubated for 18 h at 37 °C. Tryptic peptides were acidified with TFA (0.1% (v/v) final concentration), desalted with POROS R2 resin (Applied Biosystems, CA, EUA), and packaged in micropipette tips (Millipore, Bedford, USA). Desalted peptides were dried and suspended in 10 μl of 0.1% formic acid, and aliquots corresponding to 0.5 μg/μl were separated for mass spectrometry analysis.

### Mass spectrometry

The tryptic digests were analyzed by reversed-phase nanochromatography coupled to high-resolution nanoelectrospray ionization mass spectrometry. Chromatography was performed using a Dionex Ultimate 3000 RSLCnano system coupled to the HF-X Orbitrap mass spectrometry (Thermo Fischer Scientific, CA, EUA). Samples (1 μg per run) were initially applied to a 2 cm guard column, followed by fractionation on a 25.5 cm PicoFritTM Self-Pack column (New Objective) packed with 1.9 μm silica, ReproSil-684 Pur 120 Å C18-AQ (Dr. Maisch, Germany). Samples were loaded in 0.1% (v/v) formic acid (FA) and 2% acetonitrile (ACN) onto the trap column at 2 μL/min, while chromatographic separation occurred at 200 nL/min. Mobile phase A consisted of 0.1% (v/v) FA in water, while mobile phase B consisted of 0.1% (v/v) FA in ACN. Peptides were eluted with a linear gradient from 2 to 40% eluent B over 32 min, followed by up to 80% B in 4 min. The lens voltage was set to 60 V. Full-scan MS mode was acquired with a resolution of 60,000 (FWHM at m/z 200 and AGC set to 3 × 10^6^). Up to 20 of the most abundant precursor ions from each scan (m/z 350–1400) were sequentially subjected to fragmentation by HCD. Fragment ions were analyzed at a resolution of 15,000 using an AGC set to 1 × 10^5^. Data were acquired using Xcalibur software (version 4.2.47).

### Proteomic computational analysis

The raw data files were processed and quantified using PatternLab for Proteomics software [43] (version 4.0). Peptide sequence matching (PSM) was performed using the Comet algorithm against the protein-centric human database neXtProt [44] plus the SARS-CoV-2 reference proteome from UniProt [45] under ID UP000464024, both downloaded March 29, 2021. A target-decoy strategy was employed. The search parameters were tryptic and semitryptic peptides, with masses between 500 and 5000 Da, up to 2 lost cleavage sites; modifications: carbamidomethylation (Cys), oxidation (Met), and initial tolerance of 40 ppm for precursor ions. PSMs were filtered using the Search Engine Processor (SEPro) module, and identifications were grouped by the number of enzymatically cleaved ends, resulting in two distinct subgroups. For each result, the scores for each metric (XCorr, DeltaCN, and ZScore) were used to generate a Bayesian discriminator, accepting up to a 1% false discovery rate (FDR), estimated by the number of decoy sequence IDs. The results were further filtered to accept only PSMs with a mass error less than 5 ppm and protein identifications supported by two or more independent identifications. Proteins identified by a single spectrum (1 hit wonder) with XCorr below 2 were excluded. The final list of identified peptides and mapped proteins for all samples was reported. The list of resulting peptides from shotgun proteomics was used for alignment with the sequences of human endogenous retrovirus K113 (https://www.ncbi.nlm.nih.gov/nuccore/NC_022518.1). The alignment was carried out with the NCBI/BLAST database through the Protein Blast — BlastP algorithm. Alignments with identity and coverage equal to or greater than 50% were considered. Detailed information about the proteins that aligned with the peptides can be obtained from the UniProtKB SwissProt database (https://www.uniprot.org/uniprot/?query=Human+endogenous+retrovirus+K113&sort=score).

### Elisa

Blood samples were collected in ACD-containing syringes, and plasma was obtained by serial centrifugation. Whole-blood samples were centrifuged at 150 RCF/20 min/25 °C to obtain platelet-rich plasma (PRP), then 500 RCF/20 min/25 °C to obtain platelet-poor plasma (PPP), and finally 2500 RCF/20 min/25 °C to obtain platelet-free plasma, which was then aliquoted into 1 mL samples and conditioned at −80 °C. Commercial ELISA (R&D Systems, MN, USA) and Multiplex (BioRad, CA, EUA) kits were used to measure cytokines, chemokines, and coagulation markers.

### Flow cytometry

Whole blood samples were incubated for 10 min with FACS lysing buffer (BD Biosciences) and centrifuged at 400 RCF for 15 min, and the supernatant was discarded. Cells were resuspended in HEPES-Tyrode (HT) buffer (10 mM HEPES, 137 mM NaCl, 2.8 mM KCl, 1 mM MgCl_2_.6H2O, 12 mM NaHCO_3_, 0.4 mM Na_2_HPO_4_, 5.5 mM glucose, 0.35% BSA [pH 7.4]). Monocytes were labeled with fluorescein isothiocyanate (FITC)-conjugated anti-CD16, phycoerythrin (PE)-conjugated anti-TF, and peridinin-chlorophyll (PerCP)-conjugated anti-CD14 (BD Pharmingen); FITC-conjugated anti-CD38, PE-conjugated anti-CD11b, and PerCP-conjugated anti-CD14; or FITC-conjugated anti-HLA-DR, PerCP-conjugated, anti-CD14 and allophycocyanin (APC)-conjugated anti-CD83 (BD Pharmingen). Lymphocytes were labeled with FITC-conjugated anti-CD3, PE-conjugated anti-CD4, and APC-H7-conjugated anti-CD8 or with FITC-conjugated anti-CD11b, PE-conjugated anti-CD25, PE-Cy5-conjugated anti-CD38, and APC-H7-conjugated anti-CD8. B cells were labeled with FITC-labeled anti-CD38, PE-conjugated anti-CD19, PerCP-conjugated anti-CD20, and APC-H7-conjugated anti-CD27. NK cells were labeled with FITC-conjugated anti-CD107, PE-conjugated anti-CD11b, PE-Cy5-conjugated anti-CD56, APC-conjugated anti-CD3, and APC-H7-conjugated anti-CD27. Neutrophils were labeled with FITC-conjugated anti-myeloperoxidase (MPO) and PE-conjugated anti-CD11b. Cells were incubated with antibodies for 30 min at room temperature and fixed with 4% paraformaldehyde. Cells labeled with each antibody separately were used for appropriate color compensation, and isotype-matched IgG conjugated with the same fluorochromes was used as the negative control. Lymphocytes, monocytes, and neutrophils were recognized by their characteristic forward and side scatter and expression of specific surface markers, as shown in Supplementary Fig. 5. A flow cytometry (BD FACSCalibur) was used to acquire 2000 to 5000 gated events. Acquired data were further analyzed using FlowJo software.

### Cell, virus, and experimental infection

Human lung epithelial cells (Calu-3) and African green monkey kidney cells (Vero E6) were cultured in high glucose DMEM complemented with 10% fetal bovine serum (FBS), 100 U/mL penicillin, and 100 μg/mL streptomycin (P/S) at 37 °C in a humidified atmosphere with 5% CO_2_. Human primary monocytes were obtained after 3 h of plastic adherence of peripheral blood mononuclear cells (PBMCs). PBMCs were isolated from healthy donors by Ficoll density gradient centrifugation. PBMCs (2 × 10^6^ cells) were plated onto 48-well plates in RPMI-1640 without serum for 2 to 4 h. Nonadherent cells were removed, and the remaining monocytes were maintained in DMEM with 5% human serum (HS) and P/S. The purity of human monocytes was above 95%, as determined by flow cytometric analysis (FACScan; Becton Dickinson) using anti-CD3 and anti-CD16 monoclonal antibodies.

SARS-CoV-2 (GenBank # MT710714) was expanded in Vero E6 cells at an MOI of 0.01. All procedures related to virus culture were handled in a biosafety level 3 (BSL3) multiuser facility according to WHO guidelines (https://www.who.int/publications/i/item/WHO-WPE-GIH-2021.1). Virus titers were determined as plaque forming units (PFU)/mL. Virus stocks were kept in −80 °C ultralow freezers.

Infection was performed with SARS-CoV-2 at an MOI of 0.01 (monocytes) or 0.1 (Calu-3) in low (monocytes) or high (Calu-3) glucose DMEM without serum. After 1 h, the cells were washed and incubated with complete medium treatments. After 24 h (monocytes) or 48 h (Calu-3), the culture supernatant was harvested for HERV-K quantification.

### Statistics

The assays were performed blinded by one professional, codified, and then read by another professional. All experiments were carried out at least three independent times, including a minimum of two technical replicates in each assay. Prism GraphPad software 9.3.1 was preferentially used to generate the datasets. One-way analysis of variance (ANOVA) was used to compare differences among 3 or more groups following a normal (parametric) distribution, and Tukey’s post hoc test was used to locate the differences between the groups; alternatively, Friedman’s test (for nonparametric data) was used with Dunn’s post hoc test. Spearman correlation was used for comparison of curves, as well as angular and linear comparisons between discharged and deceased patients. Logistic regression was used to analyze HERV-K levels as a function of survival and early mortality. All *p* values < 0.05 were considered statistically significant.

## Results

### Human endogenous retrovirus K is transcriptionally active in the lower respiratory tract of critically ill COVID-19 patients

**Fig. 1:**
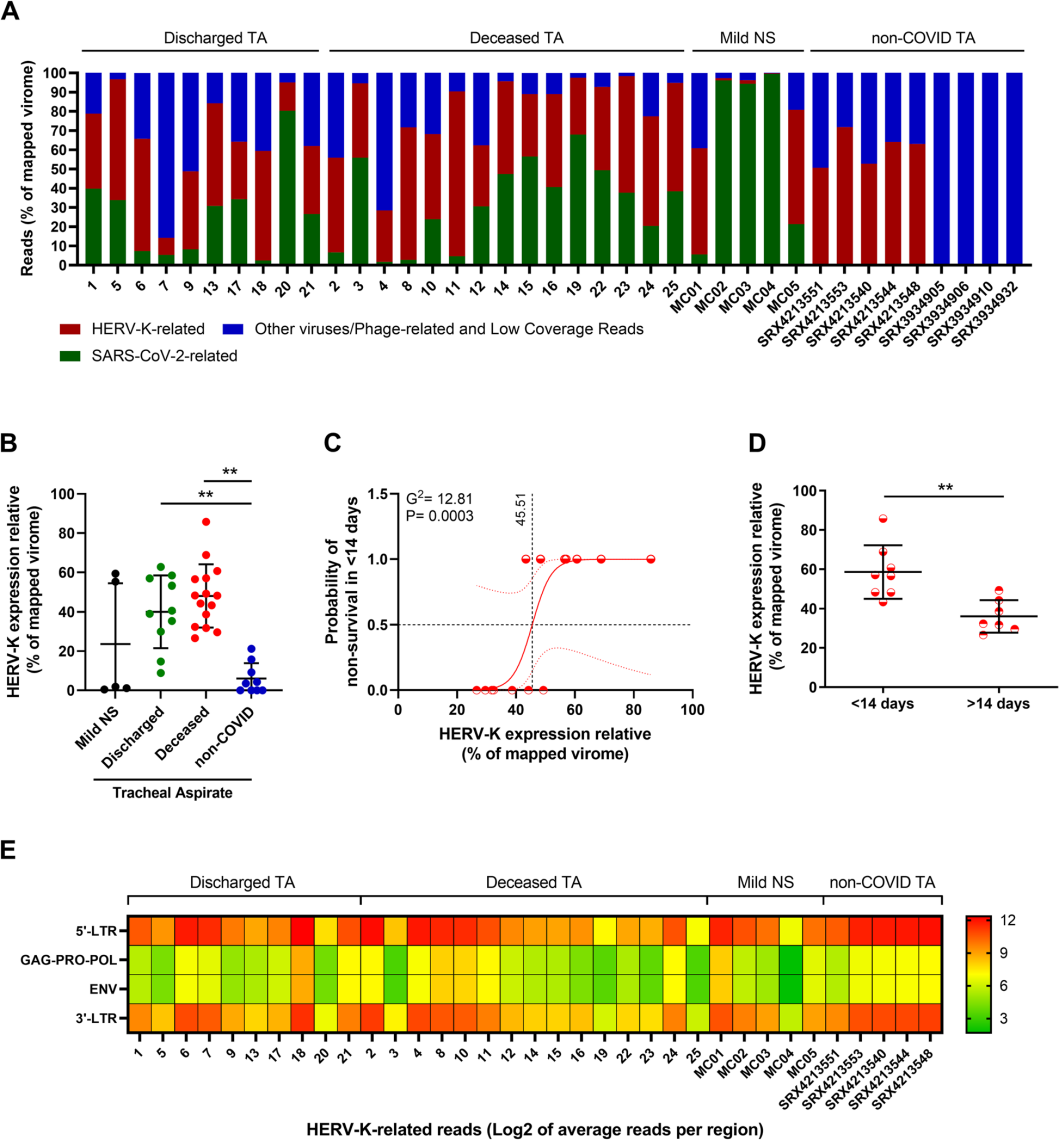
Differential overexpression of HERV-K transcripts in the lower respiratory tract of critically ill COVID-19 patients is associated with early mortality. RNA sequencing of tracheal aspirates (TA) from severe cases (Supplementary Table 1) and nasopharyngeal swabs (NS) from mild cases^17^ was performed on the MGI-2000 RNA-seq platform, and high-quality sequences (Q ≥ 30) were selected for downstream analysis. **A** Percentage of virus-related reads in the mapped virome from the TA of severe COVID-19 patients, from the NS from COVID-19 mild cases, and from non-COVID TA Sequence Read Archive (SRA) (# SRX4213540, SRX4213544, SRX4213548, SRX4213551, SRX4213553, SRX3934905, SRX3934906, SRX3934910, and SRX3934932). **B** The percentage of HERV-K-related reads in the mapped virome from the TA of discharged and deceased severe COVID-19 patients compared to NS and non-COVID TA (# SRX4213540, SRX4213544, SRX4213548, SRX4213551, SRX4213553, SRX3934905, SRX3934906, SRX3934910, and SRX3934932). **C** Logistic regression analysis between HERV-K expression and odds of early (< 14 days) mortality in deceased COVID-19 patients. Red dotted lines represent the 95% CI, while black dotted lines mark the intersection where data in x-axis represent 0.5 (50%) probability. Insert receiver operating characteristic (ROC) curve for the prediction of early (< 14 days) mortality in deceased COVID-19 patients based on HERV-K expression. **D** HERV-K expression in TA over time (days) from ICU admission to death. **E** Heatmap of absolute HERV-K read counts for TA from severe COVID-19 patients, NS from mild cases and for the non-COVID TA with HERV-K presence. **= *p* < 0.01

From March to December 2020, we prospectively included 25 critically ill COVID-19 patients requiring IMV with a median age of 57 years and presenting with the most common COVID-19 symptoms and comorbidities (Supplementary Table 1). Patients displayed high SARS-CoV-2 RNA levels (median of 10^6^ copies/mL), laboratory markers of systemic inflammation and coagulopathy (because of elevated plasma levels of C-reactive protein [CRP] and D-dimer, respectively), and a case fatality ratio of 60% (Supplementary Table 1). Due to the IMV, the tracheal aspirate (TA) was the sample source to perform SARS-CoV-2 RNA quantification and virome analysis. The TA of 70% of these patients had higher SARS-CoV-2 RNA levels than other samples from the lower respiratory tract [37] (Supplementary Fig. 1A). RNA content from TA was unbiased sequenced and rendered an average of 2 × 10^7^ genomic reads, of which up to 4% were viral-related (Supplementary Fig. 1B); from those reads, 30 ± 22% (mean ± SD) were linked to SARS-CoV-2 (Fig. 1A). For further comparisons, unbiased sequences from nasopharyngeal swabs (NS) and TA from non-COVID patients (obtained from Sequence Read Archive (SRA)) were included in our study (Supplementary Fig. 1B). After enriching new coronavirus sequences (Supplementary Table 2), we found that cases were phylogenetically related to the emerging clades 19A (16%), 20A (12%), and 20B (72%) (Supplementary Fig. 1 C and D), reconfirming that the entire cohort was composed of COVID-19 patients.

In addition to SARS-CoV-2, human endogenous retrovirus K (HERV-K; also known as HML-2) sequences were detected in the TA from COVID-19 patients at a proportion of 45 ± 17% (mean ± SD) of the virome (Fig. 1A and Supplementary Table 3). In the TA of critically ill COVID-19 patients, the detection of other viral sequences with low coverage (approximately 25%) and limited depth (less than 10x) was considered of minor importance (Fig. 1A). In some non-COVID TAs, respiratory viruses (influenza and parainfluenza) were detected (Fig. 1A, among the blue bars).

HERV-K was fivefold more present in the virome of TA from COVID-19 patients under IMV than in NS (Fig. 1B and Supplementary Table 3). Although the comparison between lower (TA) and upper (NS) respiratory tract samples may be imprecise, HERV-K RNA levels were higher in the TA from COVID-19 patients than in non-COVID-19 patients (Fig. 1 A and B). The data from SRA indicate that HERV-K may be found in the lower respiratory tract of some patients with other illnesses (Fig. 1B). To verify the correlation between HERV-K levels and the outcome of severe COVID-19 patients, we assessed the probability of survival in those patients. We found that HERV-K expression correlated with the probability of early death using logistic regression (Fig. 1C) and the Mann–Whitney test (Fig. 1D), reinforcing that HERV-K is associated with severe COVID-19 illness. Although there was a tendency to have higher HERV-K levels in deceased patients than in discharged patients, logistic regression was not statistically significant (Supplementary Fig. 2A). Among this study population, no statistically significant association was found between HERV-K RNA levels and days from COVID-19 onset, age, sex, or SARS-CoV-2 RNA levels (Supplementary Fig. 2 B–E).

Because thousands of loci in the human genome are associated with HERV-K [46], we searched for correlations between the HERV-K transcript consensus described here and active HERV-K loci in the human genome. Most often, sequences from HERV-K structural genes were expressed from different chromosomal regions, suggesting the activation of otherwise silent genes (Table S3 and Supplementary Fig. 3). Although the endogenous retrovirus consensus sequences detected in the critically ill COVID-19 patients from this study were evaluated for all known HERVs — to double-check their origins — they were phylogenetically related to HERV-K (Supplementary Fig. 3). Indeed, critically ill COVID-19 patients differentially expressed HERV-K-associated structural genes, *gag-pro-pol* and *env* transcripts, in the lower respiratory tract compared to the upper respiratory tract of mild COVID-19 patients and the lower respiratory tract of non-COVID-19 patients (Fig. 1E).

As another layer of results for the detection of HERV-K in the TA, shotgun proteomics was performed in samples from all patients. To trace similarities with the HERV-K proteome, we compared the peptides from the TA human proteome and HERV-K proteins Gag, Pro, Pol, Env, and Rec (UniProt IDs # P62684, P63121, P63132, Q902F9 and P61574, respectively) through BlastP (NCBI/BLAST). While we did not identify HERV-K proteotypic peptide signatures, we accepted BlastP matches of peptides from 20 to 47 amino acids with at least 10 amino acids and a minimum of 60% sequence identity and 80% coverage, assuming that the diversity of HERV-K peptides is not completely cataloged. With this approach, we identified a total of 29 nonredundant alignments of peptides in deceased patients and 14 peptides in discharged patients (Supplementary Fig. 4 and Supplementary files 1 and 2).

### HERV-K is also detected in the peripheral blood of COVID-19 patients

**Fig. 2:**
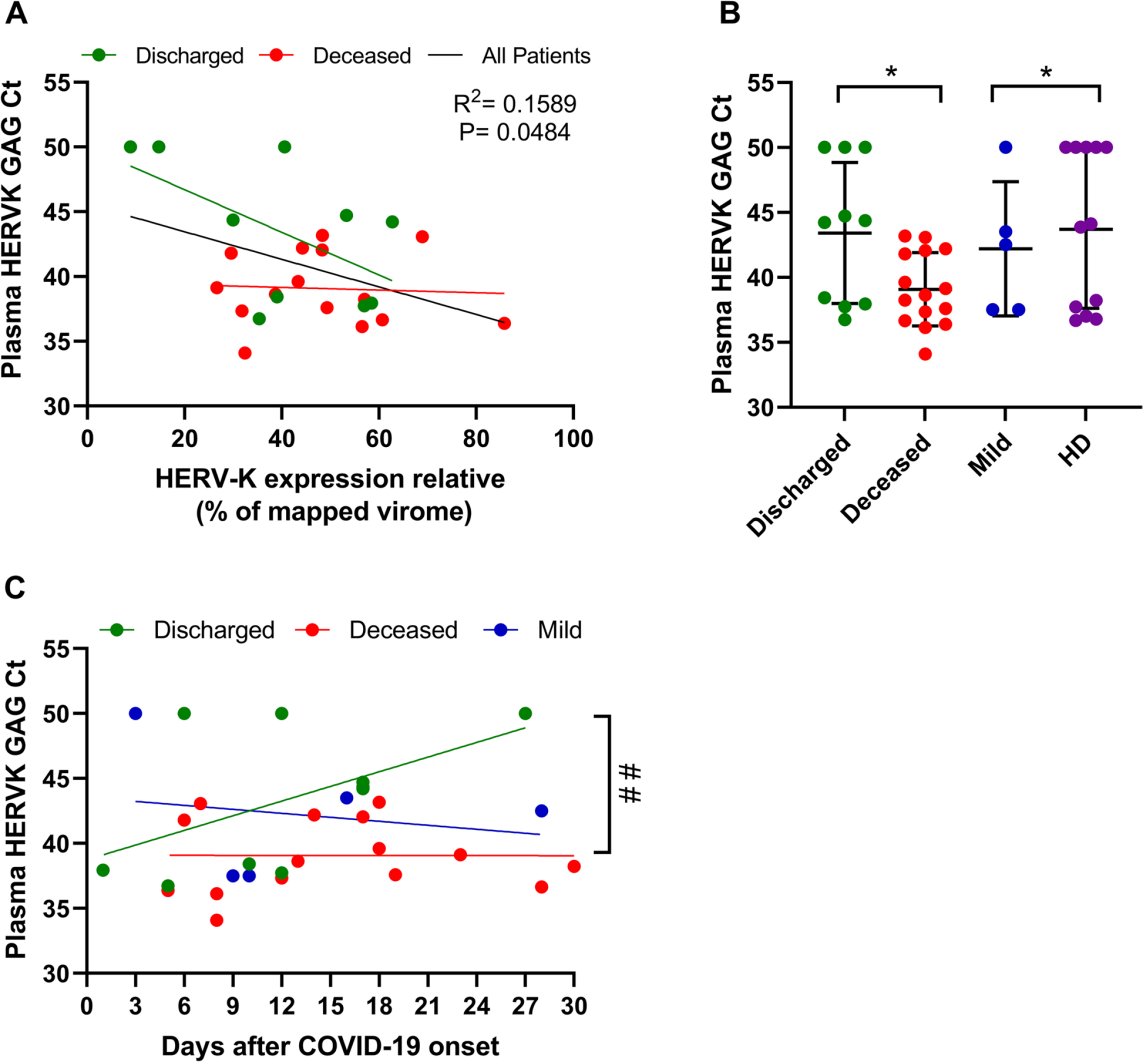
Presence of HERV-K transcripts in the plasma of severe COVID-19 patients. **A** The fraction of the HERV-K virome was compared to the results of real-time RT-qPCR to detect HERV-K GAG (Ct values) in plasma from those patients. **B** Plasma samples from severe cases (Supplementary Table 1) and from healthy donors (HD) were evaluated for the presence of HERV-K GAG by RT-qPCR. Samples with Ct values below 50 were considered positive for HERV-K. **C** HERV-K levels in the plasma of patients presented as a function of days since COVID-19 onset. A statistically significant (*p* < 0.05) difference between linear coefficients is represented by #. *= *p* < 0.05

We next sought to determine the presence of HERV-K in the plasma of COVID-19 patients by quantifying its *gag* transcripts because of its specificity to HERV-K [38]. The high levels of HERV-K in the virome of TA correlated with lower cycle threshold (Ct) values in the plasma from those patients (Fig. 2A). Indeed, HERV-K *gag* was more likely to be detected, with Ct values < 50, in the plasma of patients who died than patients who were discharged, mild COVID cases, or healthy donors (HD) (Fig. 2B), independent of the day of COVID-19 onset (Fig. 2C).

### HERV-K is associated with immune and hematologic alterations during severe COVID-19

**Fig. 3:**
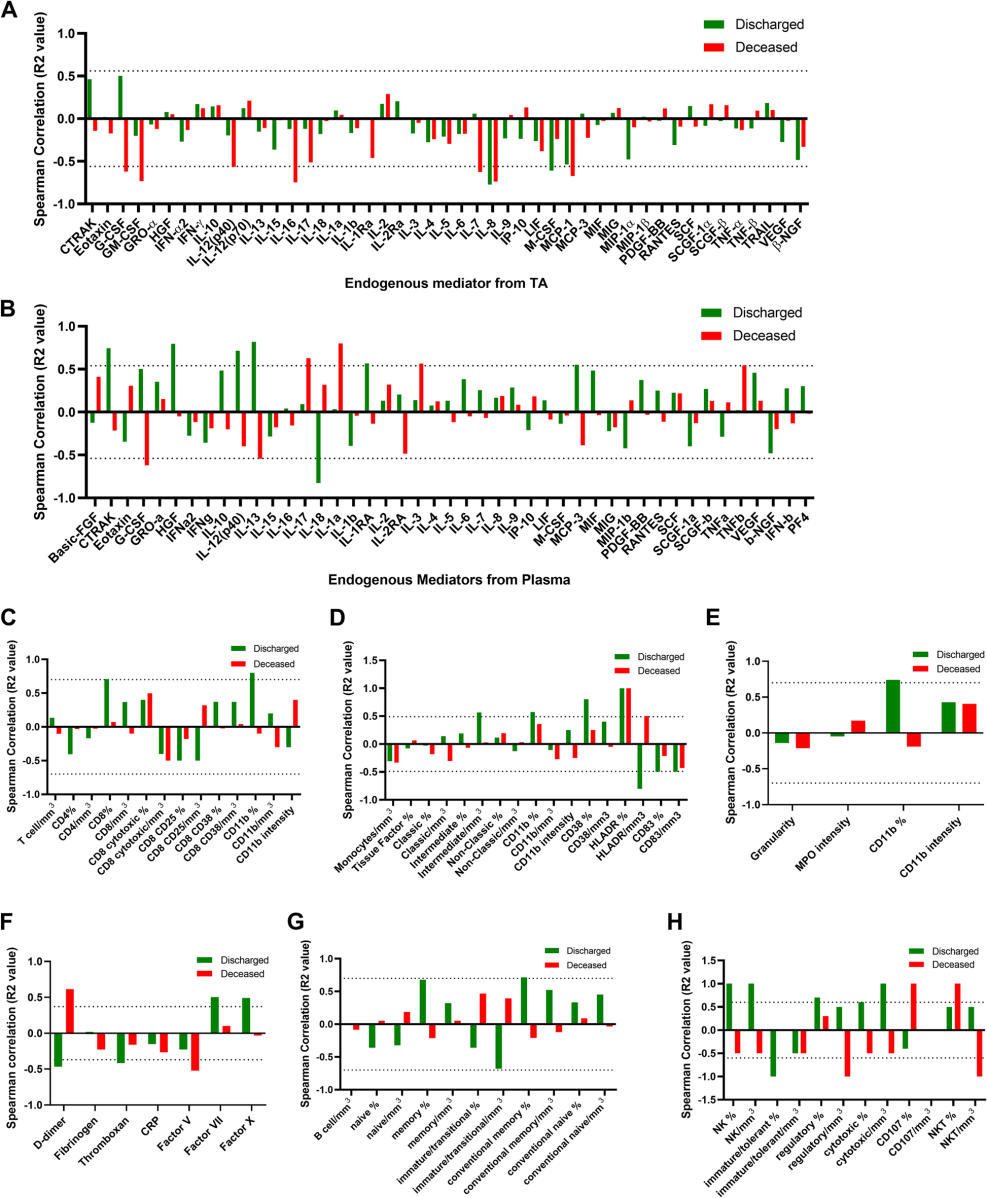
Spearman correlation between HERV-K and severity markers in COVID-19 patients. Endogenous mediators in the TA (**A**), in the plasma from peripheral blood (**B**), T cells (**C**), monocytes (**D**), neutrophils (**E**), coagulation markers in the plasma (**F**), B cells (**G**), and natural killer (NK) cells (**H**) from peripheral blood were plotted as a function of HERV-K expression. Spearman correlation R2 was plotted, and statistical significance with *p*-values < 0.05 is presented by the bars that cross the dotted lines. Gate strategy for immune cell profiling is presented in Supplementary Fig. 5

**Fig. 4:**
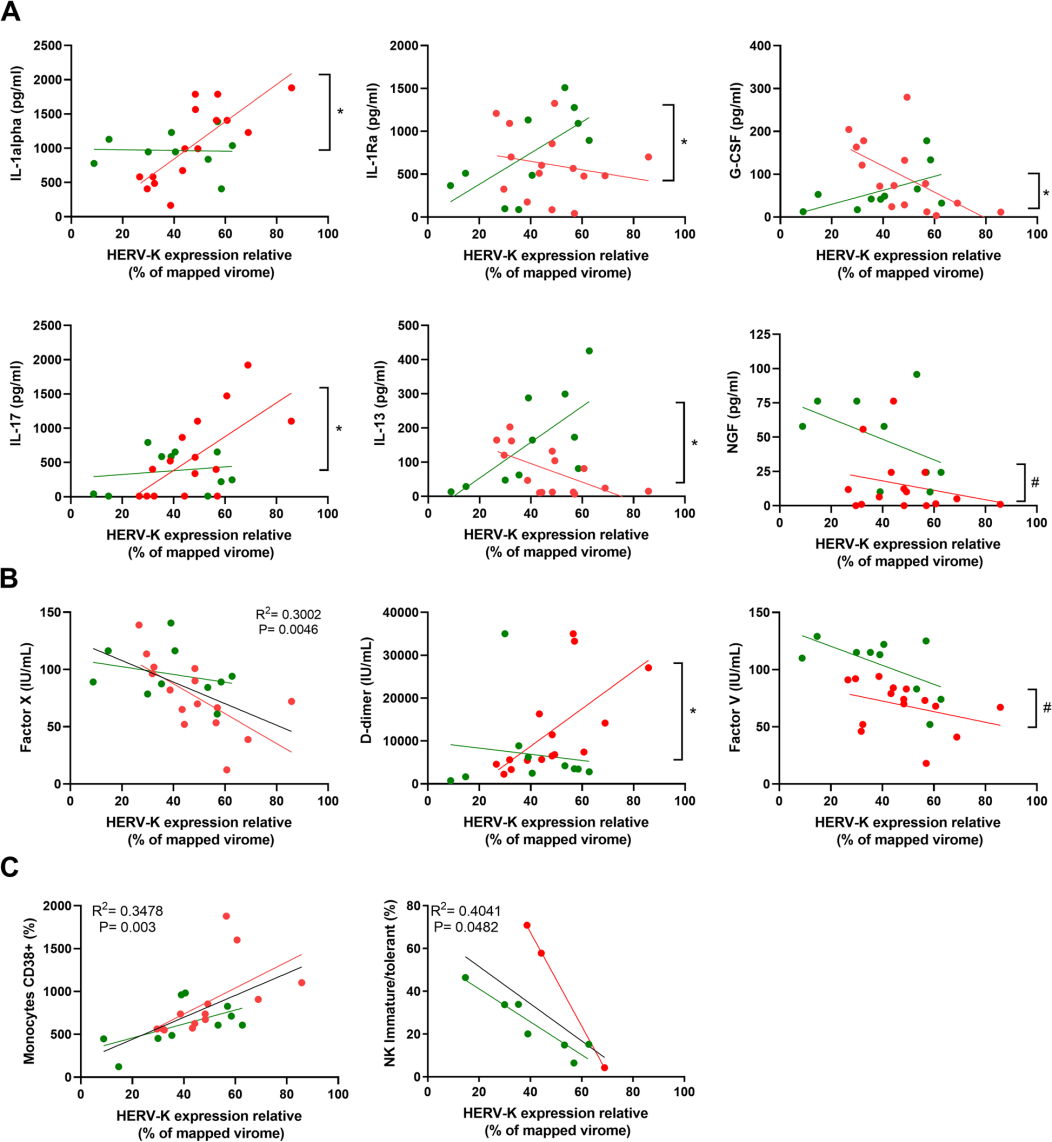
HERV-K levels correlate with immune activation and coagulopathy in a patient outcome-dependent manner. HERV-K levels are presented as the function of **A** cellular survival/differentiation factors or interleukins, **B** clotting or fibrinolysis cascade markers, and **C** immune cells. These are the statistically significant analyses from Fig. 3 (panels in **A** and **B** derived from Fig. 3 A and C, respectively; panels in **C** derived from Fig. 3 D and H). Patients and regression lines are highlighted in green for discharged and in red for deceased patients. Regression lines in black indicate statistical significance when combining both discharged and deceased patients. Statistically significant (*p* < 0.05) differences between linear or angular coefficients are represented by # or *, respectively

We next examined a possible correlation between HERV-K levels in TA with immune modulation and/or coagulopathy. For this purpose, Spearman correlation analysis for levels of cytokines, coagulation factors, and immune cell counts was scored in deceased and discharged patients (Fig. 3). As a general tendency for the endogenous mediators, HERV-K reduced their levels in the TA (Fig. 3A) and favored inflammation in the peripheral plasma (Fig. 3B). To be conservative when assuming statistical significance, we additionally performed regression analysis for those markers that passed Spearman correlation, evaluating differences in angular and/or linear coefficients (Fig. 4). HERV-K levels in deceased patients were positively associated with the proinflammatory markers IL-1alpha and IL-17 (Fig. 4A). Regarding the regulatory molecules IL-1Ra and IL-13, the results from deceased and discharged patients were dichotomic as a function of HERV-K levels (Fig. 4A), favoring regulatory signals in critically ill survivors. Moreover, HERV-K levels were negatively associated with two survival/growth factors for immune cells, granulocyte colony-stimulating factor (G-CSF), and nerve growth factor (NGF) (Fig. 4A).

In light of HERV-K levels, clotting factors were altered (Fig. 3C). For example, factor X consumption was higher, independent of the disease outcome (Fig. 4B). An apparent higher consumption of factor V and levels of fibrinolysis (D-dimer) occur as a function of HERV-K levels in deceased patients (Fig. 4B).

To correlate with cell-mediated immunity, specific populations were quantified by flow cytometry (Supplementary Fig. 5) and plotted as a function of HERV-K levels (Fig. 3 D–H). Monocyte activation positively correlated with HERV-K, whereas HERV-K negatively correlated with natural killer cells (Fig. 4C), suggesting a contribution to impair an adequate innate antiviral response.

### SARS-CoV-2 triggers HERV-K expression in human primary monocytes in a viral- and immune-dependent fashion

For further evidence of a causal relationship between SARS-CoV-2 and the expression of endogenous retrovirus, we experimentally infected Calu-3 cells or human primary monocytes obtained from healthy donors. The choice of these cell models was to represent, at the cellular level, two major cellular compartments affected by critically ill COVID-19. Calu-3 cells recapitulate the main replication site of SARS-CoV-2 on type II pneumocytes [47, 48]. Critically ill patients also present leukopenia in the peripheral blood [20, 49], and when circulating monocytes migrate to the infected lung [8, 50], their exposure to SARS-CoV-2 leads to an unbaled proinflammatory response culminating in necrotic cell death, such as pyroptosis, which will enhance the cytokine storm and immune cell dysfunction observed in COVID-19 patients [5, 8, 14, 16, 18, 51].

**Fig. 5:**
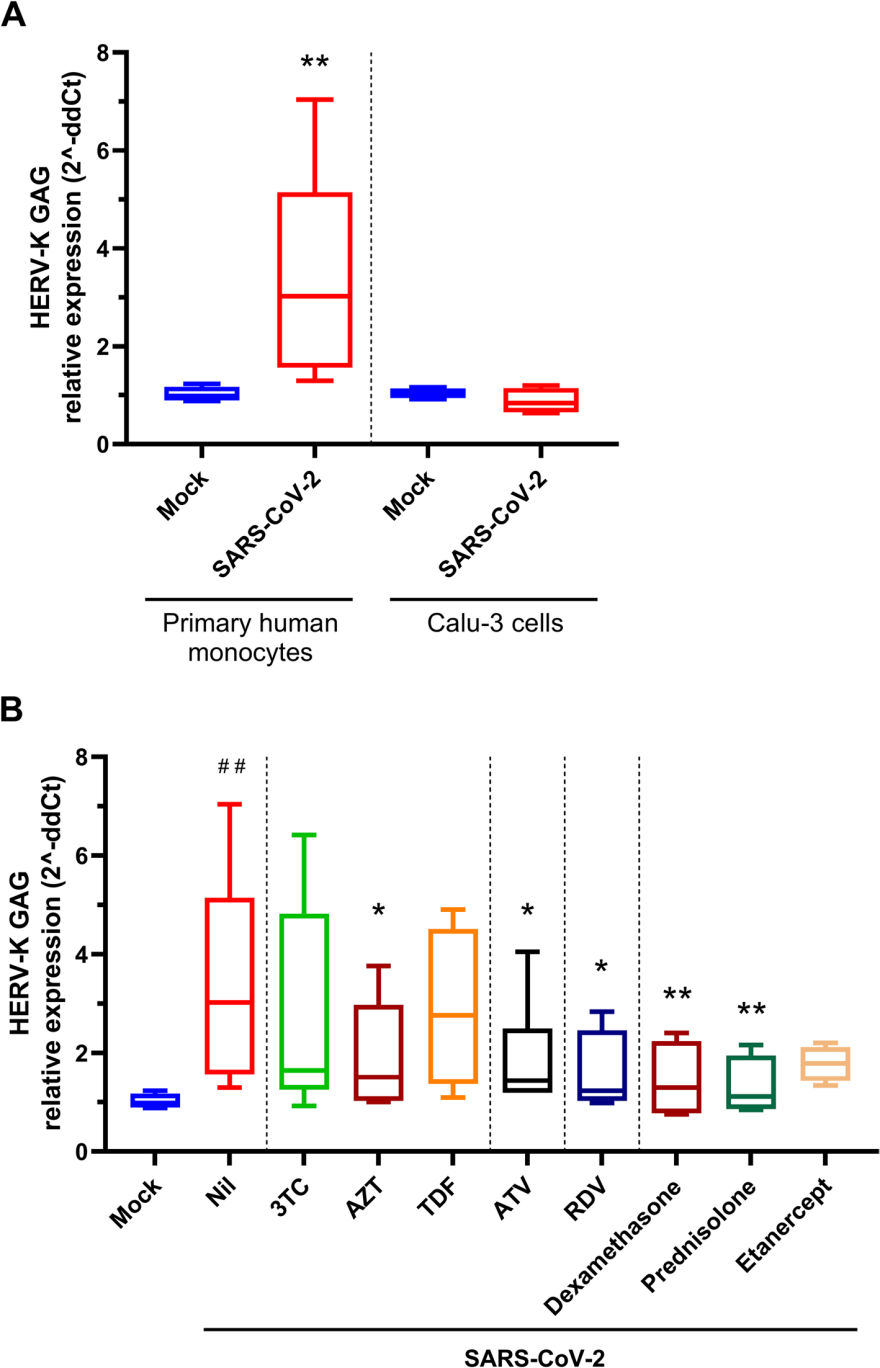
Engagement of HERV-K expression by SARS-CoV-2 infection. **A** Human primary monocytes or Calu-3 cells were infected with an MOI of 0.1. **B** Human primary monocytes or Calu-3 cells were infected with an MOI of 0.1 and treated with antivirals (10 μM each) or anti-inflammatory drugs (10 μM dexamethasone and prednisolone, 25 ng/mL etanercept). **A** and **B** At 24-h postinfection, cells were lysed, and total RNA was used to quantify HERV-K GAG and RPL19 (as a reference gene). Data are presented as relative expression following the 2^-ddCt procedure. Human primary monocytes (*n* = 5, 2 technical replicates), Calu-3 cells (*n* = 3, 2 technical replicates); *= p < 0.05; **= p < 0.01

We found that upon SARS-CoV-2 infection, HERV-K was upregulated in monocytes but not in Calu-3 cells (Fig. 5A). Next, we evaluated whether specific treatments could prevent SARS-CoV-2-dependent HERV-K expression in primary monocytes. Despite limited activity against SARS-CoV-2 [52], some HIV-1 reverse transcriptase inhibitors described to inhibit retrotransposons and HERVs [53, 54], such as lamivudine (3TC), zidovudine (AZT), and tenofovir disoproxil fumarate (TDF), prevented HERV-K expression (Fig. 5B). Atazanavir (ATV), an HIV protease inhibitor with some activity against SARS-CoV-2 [55], diminishes HERV-K expression (Fig. 5B). More notably, the anti-coronavirus drug remdesivir (RDV) impaired SARS-CoV-2-dependent HERV-K expression (Fig. 5B). Similarly, the broad steroidal anti-inflammatory drugs dexamethasone and prednisolone promoted a reduction in HERV-K expression, and the anti-TNF biopharmaceutical etanercept, despite showing some level of HERV-K expression inhibition, did not achieve statistical significance (Fig. 5B). These results confirm, at the cellular level, that SARS-CoV-2 replication and immunomodulatory properties favor HERV-K expression.

## Discussion

The SARS-CoV-2 emerging clades circulating in Brazil during 2020 (https://nextstrain.org/ncov/global?dmax=2020-12-16&dmin=2020-01-16&f_country=Brazil) were found to activate HERV-K in the lower respiratory tract of critically ill COVID-19 patients under IMV. HERV-K levels were higher in patients who died soon after the onset of illness. Endogenous retrovirus gene expression was associated with broad chromosomal activation and differential upregulation compared to non-COVID patients. In addition to the respiratory tract, HERV-K levels were also higher in the plasma of COVID-19 patients who died than in patients who were discharged and healthy donors. Increased HERV-K expression in deceased patients was associated with upregulation of proinflammatory markers, monocyte activation, and increased consumption of clotting factors. Through experimental infection in human primary monocytes, SARS-CoV-2 induced HERV-K expression, which was diminished by antivirals against COVID-19 and anti-inflammatory drugs. Our data implicate HERV-K in the physiopathology of critically ill COVID-19 patients.

Among endogenous retroviruses, HERV-K has been incorporated into the genome of the human lineage during divergence from chimpanzees [56]. Thus, it is noteworthy to find a human-specific marker associated with critically ill COVID-19 patients, as nonhuman primates are less likely to die from SARS-CoV-2 infection [57], raising the attention to a possible role of HERV-K in the dichotomy of SARS-CoV-2 severity between humans and nonhuman primates. Indeed, HERV-K detection in the respiratory tract has been associated with lung adenocarcinoma [58], as well as other types of cancer, neurological disorders, multiple sclerosis, and arthritis [59].

We found profound immunomodulation in association with HERV-K, similar to other diseases [59] and to negative clinical outcomes [60]. Likewise, HERVs have been associated with the modulation of G-CSF [61] and NGF [62] levels. As a function of HERV-K levels, regulatory/anti-inflammatory signals were also decreased in the plasma of deceased patients, such as IL-1Ra and IL-13, which antagonize IL-1-dependent stimuli and favor an allergenic-like/TH2 response, respectively [63, 64]. Interestingly, the reduction of IL-13 production is also reported by a HERV-H-LTR-derived protein, together with the inhibition of CD4 and CD8 T-cell responses [65]. Deceased patients respond to higher HERV-K levels increasing IL-17, a further proinflammatory mediator that may upregulate IL-6, CRP, and airway remodeling [65] and is upregulated by HERVs in autoimmune diseases [66].

Along with the differential expression of HERV-K genes, immunomodulation, coagulopathy, and disease severity may suggest that Gag and protease could lead to immune dysregulation [67, 68]. HERV-K reverse transcriptase may jeopardize the cell cycle of lymphocytes [69]. Protease has been associated with progressive obliterative vascular remodeling in the respiratory tract [68]. HERV-K Env may trigger cell–cell fusion, leading to epithelial to mesenchymal transition, including in the respiratory tract [58, 70]. In addition to the predictive HERV-K effects on cellular and molecular biology described above, HERV-K reverse transcriptase could favor the integration of SARS-CoV-2 genetic elements into the host cell genome [32].

In addition to the identification of HERV-K in the lower respiratory tract, we also found this endogenous retrovirus in plasma and associated it with disease fatality. This detection and HERV-associated immunomodulation are in line with HERV-W Env expression in T cells from critically ill COVID-19 patients [33] and with the direct induction of HERV-W Env protein upon in vitro SARS-CoV-2 infection of PBMCs [34]. Additionally, other endogenous retroelements have been implicated in SARS-CoV-2 infection and COVID-19 severity, as Alu retrotransposons, LINE-1 elements, HERV-H, and -FRD were identified either in in vitro or in patient sample analyses [30–32, 35, 36]. The detection of HERVs in the peripheral blood of critically ill COVID-19 patients could be a contributing factor for extrapulmonary manifestations of this new disease. HERV-K is associated with monocyte activation and is upregulated by experimental SARS-CoV-2 infection.

Our group and others have consistently demonstrated that SARS-CoV-2 replication in monocytes is nonpermissive [71–74], meaning monocytes may be infected and harbor virus genome synthesis, but do not productively produce infectious SARS-CoV-2 particles. Upon SARS-CoV-2 exposure, our group and others showed that monocytes undergo pyroptosis and release of proinflammatory factors [14, 75], which could be a positive feedback to upregulate HERV-K. By succumbing to lytic cell death, SARS-CoV-2-infected monocytes contribute to the exacerbation of inflammation associated with the cytokine storm and do not execute their function as antigen-presenting cells to orchestrate the immune response [5, 8, 14, 16, 18, 27, 51]. Importantly, remdesivir, which limits SARS-CoV-2 RNA synthesis in monocytes [74], could prevent the coronavirus-dependent enhancement of HERV-K levels, meaning that early events associated with SARS-CoV-2 infection could trigger HERV-K. At a different magnitude ATV, which is endowed with limited anti-SARS-CoV-2 major protease inhibition [55], another early event in the coronavirus life cycle could reduce HERV-K levels. The HIV reverse transcriptase inhibitor AZT reduced HERV-K expression, suggesting that SARS-CoV-2-triggered HERV-K enhancement could suffer positive feedback from the newly expressed endogenous retrovirus.

## Conclusions

Our data imply that HERV-K may be upregulated due to SARS-CoV-2 and COVID-19 inflammation. The association of HERV-K with hematological changes reinforces its contributions to the physiopathology of COVID-19 in critically ill patients and early mortality.

## Supplementary Information

**Additional file 1: Supplementary Figure 1**: Characteristics of the SARS-CoV-2 detected in the tracheal aspirates of patients under the invasive mechanical ventilation. **Supplementary Figure 2**: HERV-K expression and social-demographic indicators of the cohort. **Supplementary Figure 3**: Representative phylogenetic trees of Gag, Pol and Env of HERV-K. **Supplementary Figure 4**: Schematic representation of non-redundants BlastP alignments detected between peptides identified in tracheal aspirate proteome and HERV-K proteins. **Supplementary Figure 5**: Gate strategy for the immune profiling of severe COVID-19 patients. **Supplementary Table 1**: Demographic clinical and laboratorial aspects of the patients. **Supplementary Table 2**: Quality control of SARS-CoV-2 sequences. **Supplementary Table 3**: Quality control of the HERV-K sequences

**Additional file 2.**

**Additional file 3.**

## Data Availability

All nucleotide data sequence generated during this work is available at the NCBI GenBank, under respective accession codes informed in Supplementary Tables 2 and 3. Proteomic data is available in Supplementary files 1 and 2.
