# Supplementary material for: Human endogenous retrovirus K in the respiratory tract is associated with COVID-19 physiopathology
Source: Microbiome. 2022 Apr 22;10:65. doi: 10.1186/s40168-022-01260-9 (PMC9024070; doi:10.1186/s40168-022-01260-9)
Supplement: Supplementary file 2 — Additional file 1: Supplementary Figure 1: Characteristics of the SARS-CoV-2 detected in the tracheal aspirates of patients under the invasive mechanical ventilation. Supplementary Figure 2: HERV-K expression and social-demographic indicators of the cohort. Supplementary Figure 3: Representative phylogenetic trees of Gag, Pol and Env of HERV-K. Supplementary Figure 4: Schematic representation of non-redundants BlastP alignments detected between peptides identified in tracheal aspirate proteome and HERV-K proteins. Supplementary Figure 5: Gate strategy for the immune profiling of severe COVID-19 patients. Supplementary Table 1: Demographic clinical and laboratorial aspects of the patients. Supplementary Table 2: Quality control of SARS-CoV-2 sequences. Supplementary Table 3: Quality control of the HERV-K sequences [file 40168_2022_1260_MOESM2_ESM.docx]

**Supplementary data for:**

**Human endogenous retrovirus K in the respiratory tract is associated with COVID-19 physiopathology.**

Jairo R. Temerozo^a,b,c,d,1^, Natalia Fintelman-Rodrigues^c,d,1^, Monique Cristina dos Santos^c^, Eugenio D. Hottz^c,e^, Carolina Q. Sacramento^c,d^, Aline de Paula Dias da Silva^c,d^, Samuel Coelho Mandacaru^d,f^, Emilly Caroline dos Santos Moraes^d,f^, Monique R. O. Trugilho^d,f^, João S. M. Gesto^d^, Marcelo Alves Ferreira^d^, Felipe Betoni Saraiva^g^, Lohanna Palhinha^c^, Remy Martins-Gonçalves^c^, Isaclaudia Gomes Azevedo-Quintanilha^c^, Juliana L. Abrantes^h^, Cássia Righy^i,j^, Pedro Kurtz^i,k^, Hui Jiang^l^, Hongdong Tan^l^, Carlos Morel^d^, Dumith Chequer Bou-Habib^a,b^, Fernando A. Bozza^j,k^, Patrícia T. Bozza^c^ and Thiago Moreno L. Souza^c,d^

^a^Laboratory on Thymus Research, Oswaldo Cruz Institute (IOC), Oswaldo Cruz Foundation (Fiocruz), Rio de Janeiro, RJ, Brazil; ^b^National Institute for Science and Technology on Neuroimmunomodulation (INCT/NIM), Oswaldo Cruz Institute (IOC), Oswaldo Cruz Foundation (Fiocruz), Rio de Janeiro, RJ, Brazil; ^c^Laboratory of Immunopharmacology, Oswaldo Cruz Institute (IOC), Oswaldo Cruz Foundation (Fiocruz), Rio de Janeiro, RJ, Brazil; ^d^National Institute for Science and Technology on Innovation on Neglected Diseases (INCT/IDN), Center for Technological Development in Health (CDTS), Oswaldo Cruz Foundation (Fiocruz), Rio de Janeiro, RJ, Brazil; ^e^Laboratory of Immunothrombosis, Department of Biochemistry, Federal University of Juiz de Fora (UFJF), Minas Gerais, Brazil; ^f^Laboratory of Toxinology, Oswaldo Cruz Institute (IOC), Oswaldo Cruz Foundation (Fiocruz), Rio de Janeiro, RJ, Brazil; ^g^Instituto de Tecnologia em Imunobiológicos (Bio-Manguinhos), Oswaldo Cruz Foundation (Fiocruz), Rio de Janeiro, RJ, Brazil; ^h^Instituto de Ciências Biomédicas, Federal University of Rio de Janeiro (UFRJ), Rio de Janeiro, RJ, Brazil; ^i^Paulo Niemeyer State Brain Institute (IECPN), Rio de Janeiro, RJ, Brazil; ^j^Evandro Chagas National Institute of Infectious Diseases, Oswaldo Cruz Foundation (Fiocruz), Rio de Janeiro, RJ, Brazil; ^k^D’Or Institute for Research and Education, Rio de Janeiro, RJ, Brazil; ^l^MGI Tech Co. Ltd., Building No.11, Beishan Industrial Zone, Yantian District, Shenzhen 518083, China.

^1^J.R.T. and N.F.-R. and contributed equally to this work.

**Corresponding author line:** Thiago Moreno Lopes e Souza, Fundação Oswaldo Cruz (Fiocruz), Centro de Desenvolvimento Tecnológico em Saúde (CDTS), Instituto Oswaldo Cruz (IOC), Pavilhão 108, sala 49, Av. Brasil 4365, Manguinhos, Rio de Janeiro, RJ, Brasil, CEP 21060340; tmoreno@cdts.fiocruz.br


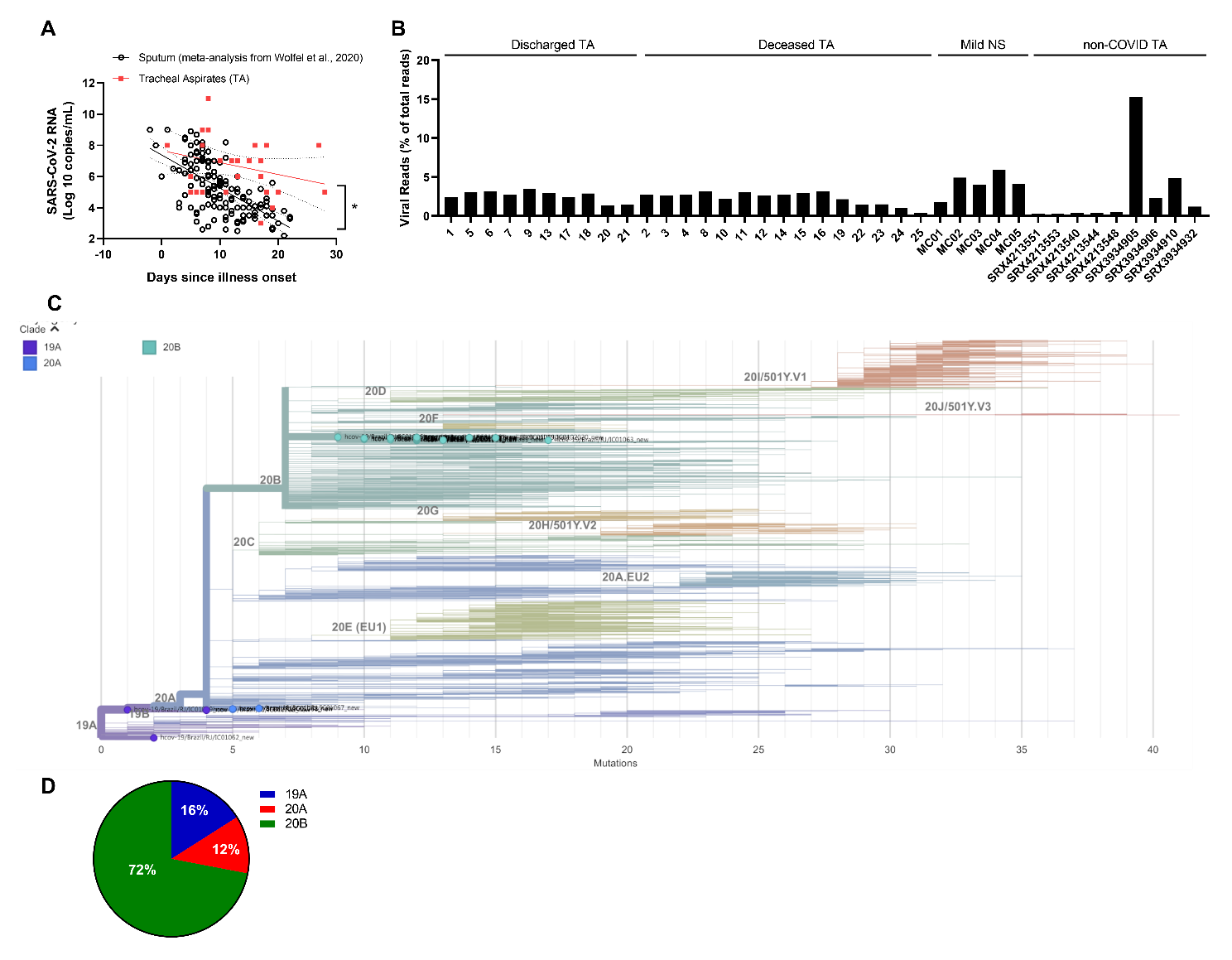


**Supplementary Figure 1** – **Characteristics of the SARS-CoV-2 detected in the tracheal aspirates of patients under the invasive mechanical ventilation. A)** Linear regression (± 95% CI) of SARS-CoV-2 RNA levels from tracheal aspirates (TA) of patients under mechanical ventilation (red), compared to meta-analysis of the sputum from Wolfel et al., 2020[15]. **B)** Percentage of virus-related reads in the transcriptome from TA of severe COVID-19 patients and from NS from COVID-19 mild cases. **B)** Phylogenetic tree of the full-length consensus SARS-CoV-2 genomes, generated with Atoplex kit using a MGI-2000 sequencer, from TA (circles) was constructed through NextClade[45] and colored according to the emerging clade default definitions. **C)** Distribution of SARS-CoV-2 emerging clades among the patients.


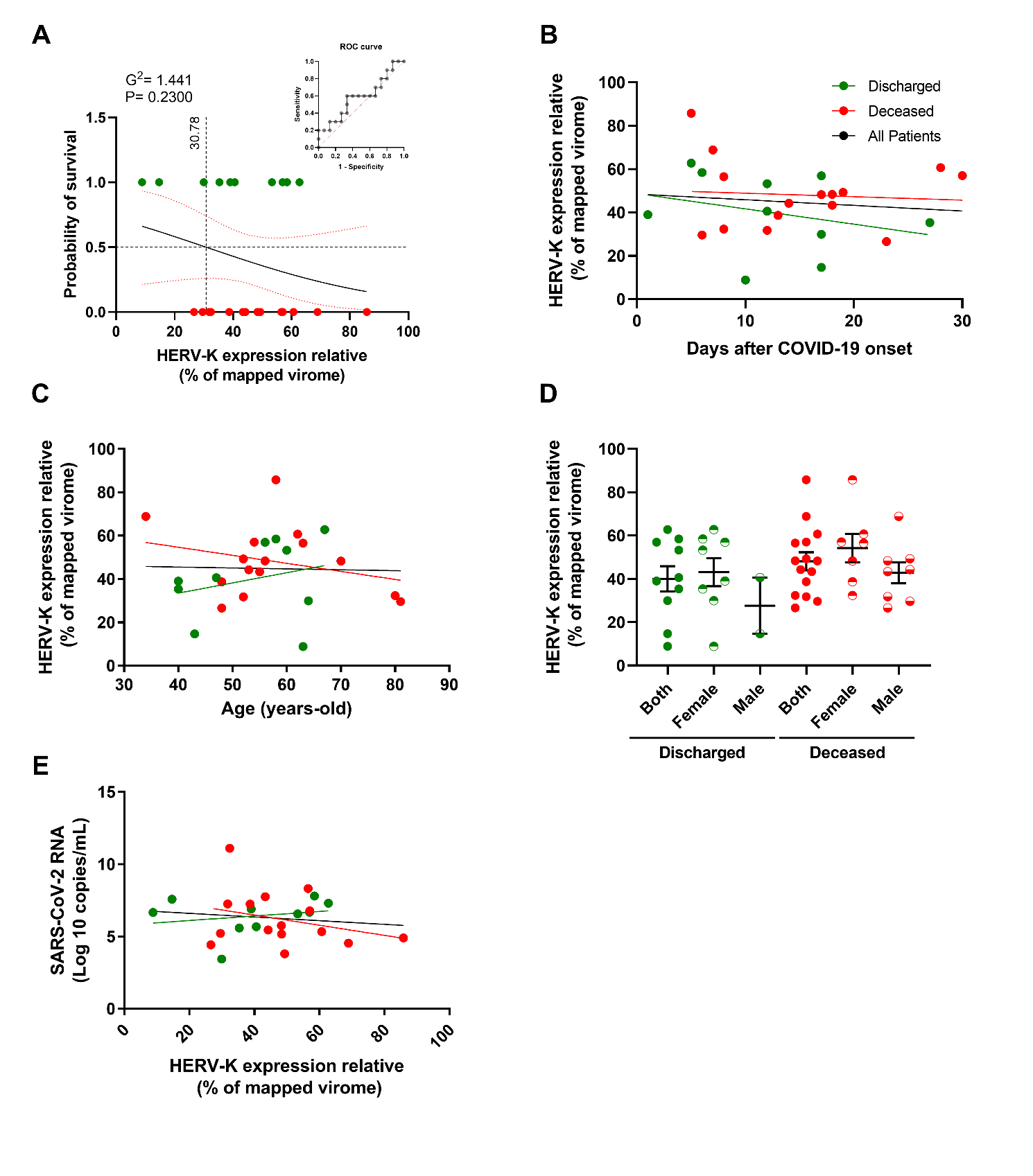
 **Supplementary Figure 2** – **HERV-K expression and social-demographic indicators of the cohort.** (A) Logistic regression between HERV-K expression and survival of severe COVID-19 patients. Red dotted lines represent the 95% CI, while black dotted lines mark the intersection where data in x axis represent 0.5 (50%) probability. Insert: Receiver operating characteristic (ROC) curve for prediction of survival of severe COVID-19 patients based on the HERV-K expression. HERV-K expression is presented as a function of **(B)** days since COVID-19 onset **(C)** age, **(D)** gender and **(E)** SARS-CoV-2 RNA levels.

**A**

**
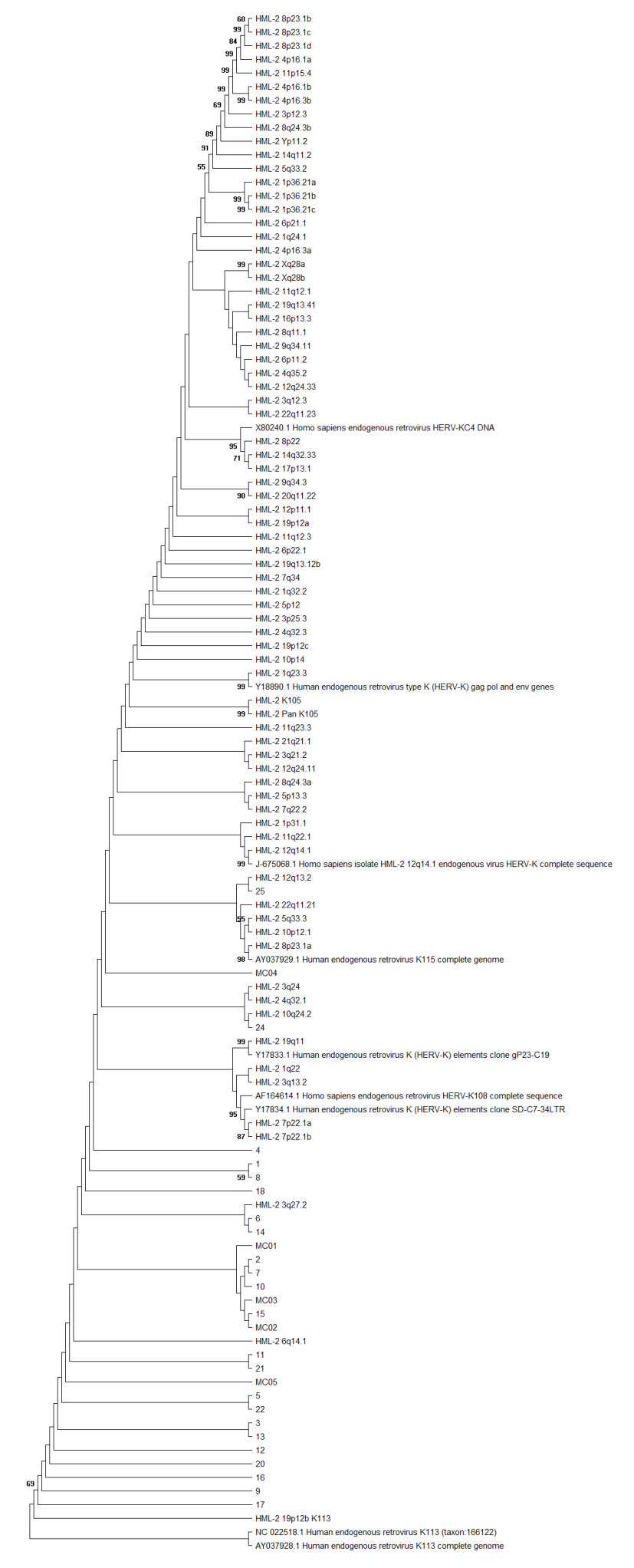
**

**B**

**
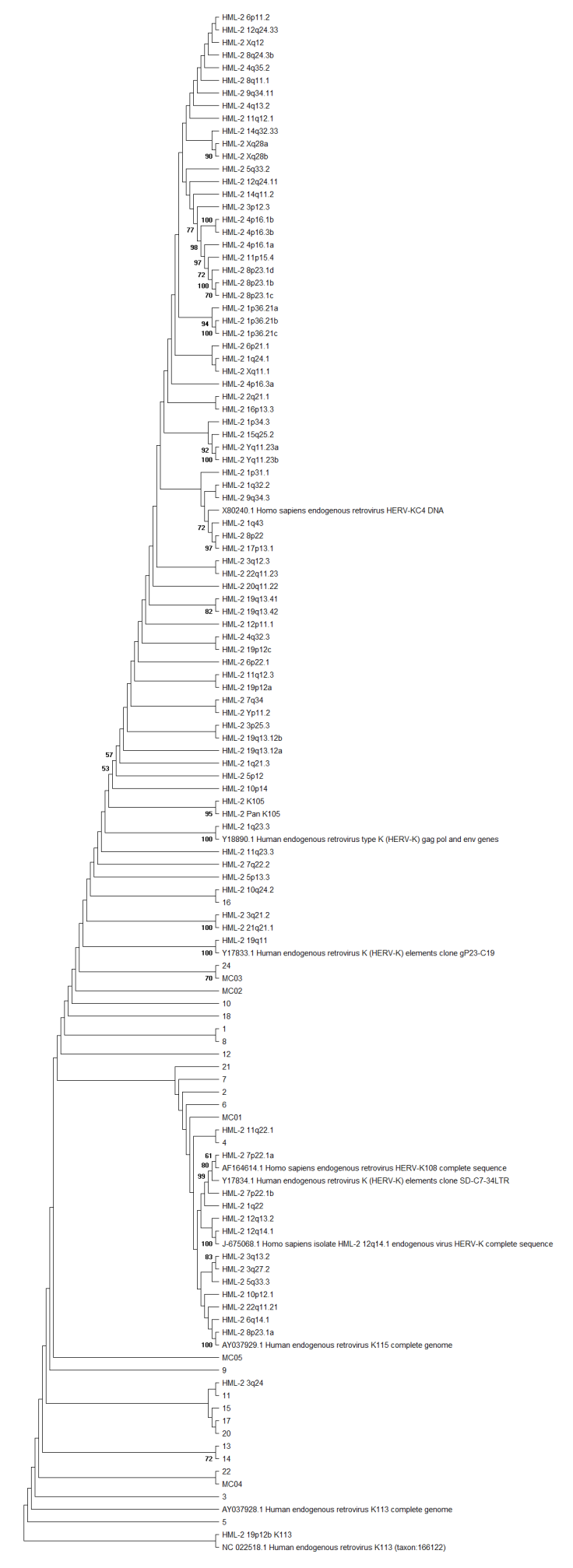
**

**C**

**
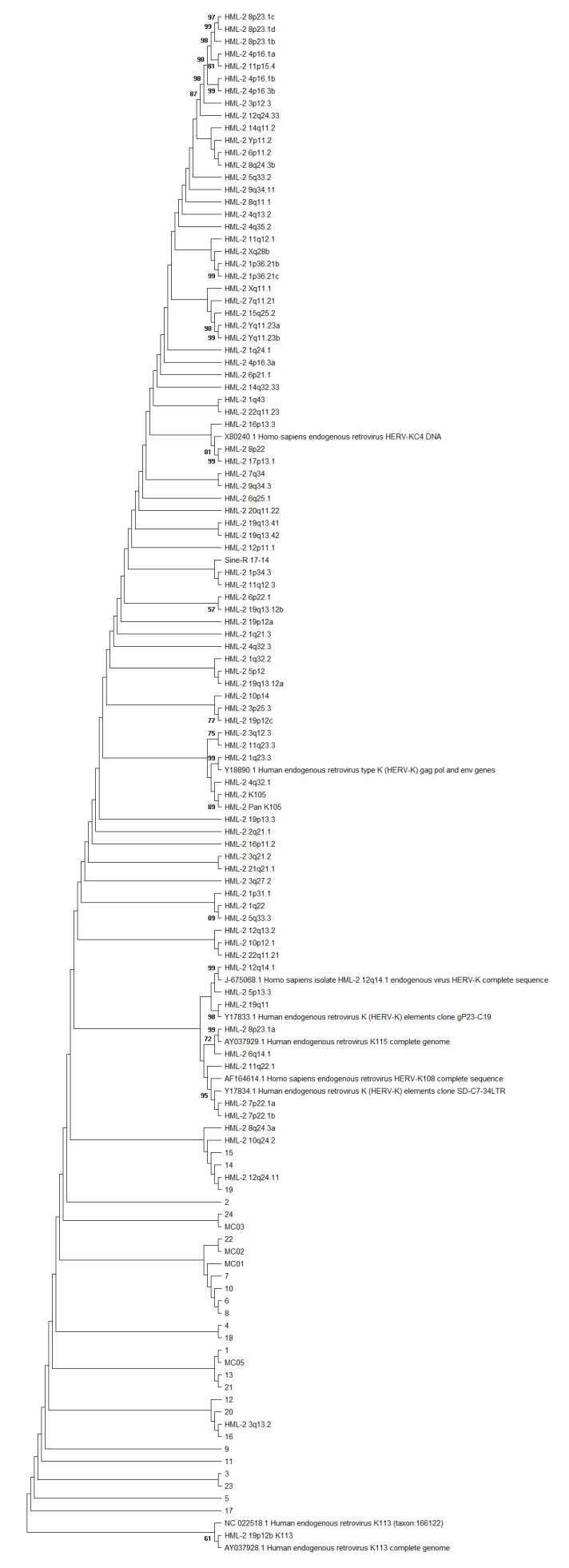
**

**Supplementary Figure 3** – **Representative phylogenetic trees of Gag, Pol and Env of HERV-K.** Representative genomes deposited in GenBank were compared with sequences from HERV-K polymerase, gag and envelope genome found in the tracheal aspirate samples from severe COVID-19 patients and nasal swabs from mild COVID-19. Three condensed phylogenetic trees rooted by reference genomes (AY037928.1 and NC 022518.1) were created with a total of 100 bootstraps. Evolutionary analyses were conducted in MEGA X[47]. **(A)** Gag evolutionary history was inferred using the maximum-likelihood method and Tamura-Nei model using a discrete Gamma distribution to model evolutionary rate differences among sites (5 categories (+*G*, parameter = 1,4126)). The tree with the highest log likelihood (-27455.55) is shown. This analysis involved 113 nucleotide sequences and there were a total of 2651 positions in the final dataset. **(B)** Pol evolutionary history was inferred using the maximum-likelihood method and General Time Reversible model using a discrete Gamma distribution to model evolutionary rate differences among sites (5 categories (+*G*, parameter = 1.1361)). The tree with the highest log likelihood (-31587.82) is shown. This analysis involved 122 nucleotide and there were a total of 2719 positions in the final dataset. **(C)** Env evolutionary history was inferred using the maximum-likelihood method and Hasegawa-Kishino-Yano model using a discrete Gamma distribution to model evolutionary rate differences among sites (5 categories (+*G*, parameter = 1.7307)). The tree with the highest log likelihood (-31664.88) is shown. This analysis involved 125 nucleotide sequences and there were a total of 2382 positions in the final dataset.


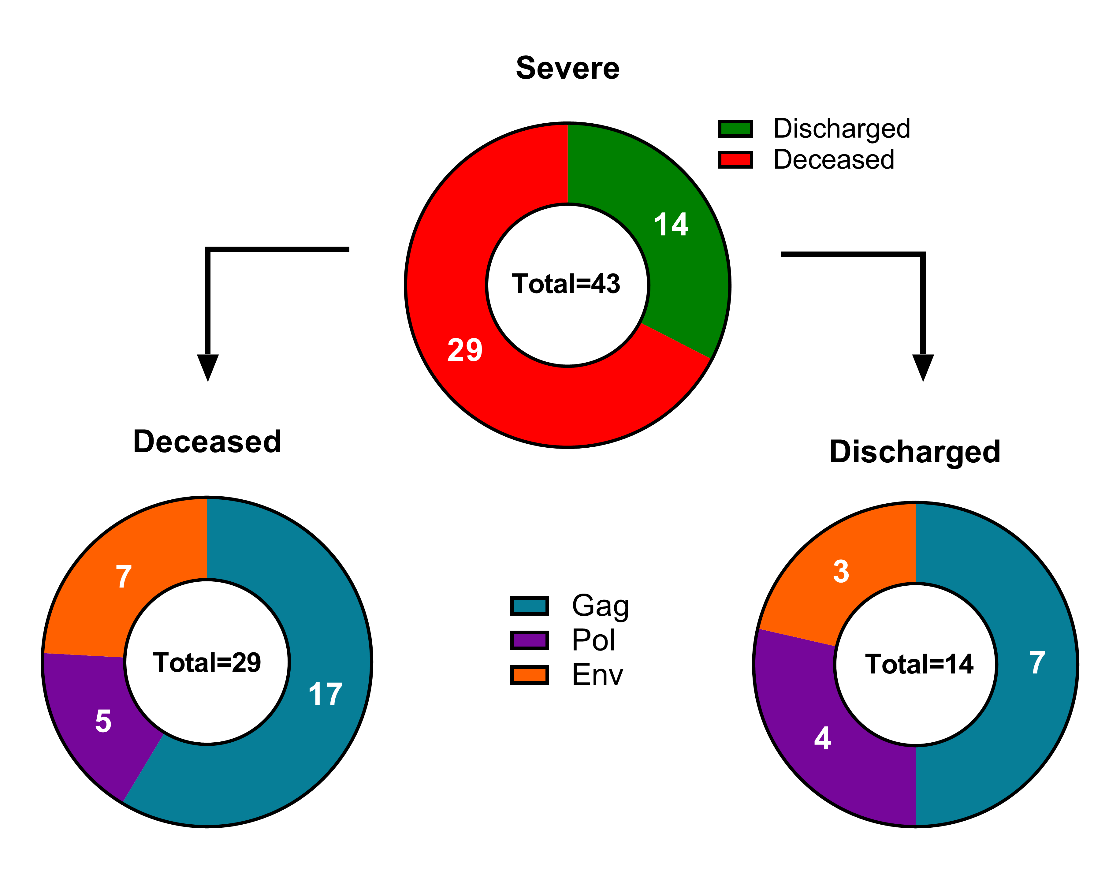


**Supplementary Figure 4** – **Schematic representation of non-redundants BlastP alignments detected between peptides identified in tracheal aspirate proteome and HERV-K proteins.** Shotgun proteomics in TA samples from all patients and, peptides from TA human proteome were compared to HERV-K proteins Gag, Pro, Pol, Env and Rec (Uniprot IDs # P62684, P63121, P63132, Q902F9 and P61574, respectively) through BlastP (NCBI/BLAST), accepting matches of peptides from 20 to 47 amino acids with at least 10 amino acids, and a minimum of 60% of sequence identity and 80% of coverage. Non-redundant alignments in HERV-K proteins were identified in Gag, Pol and Env.

**
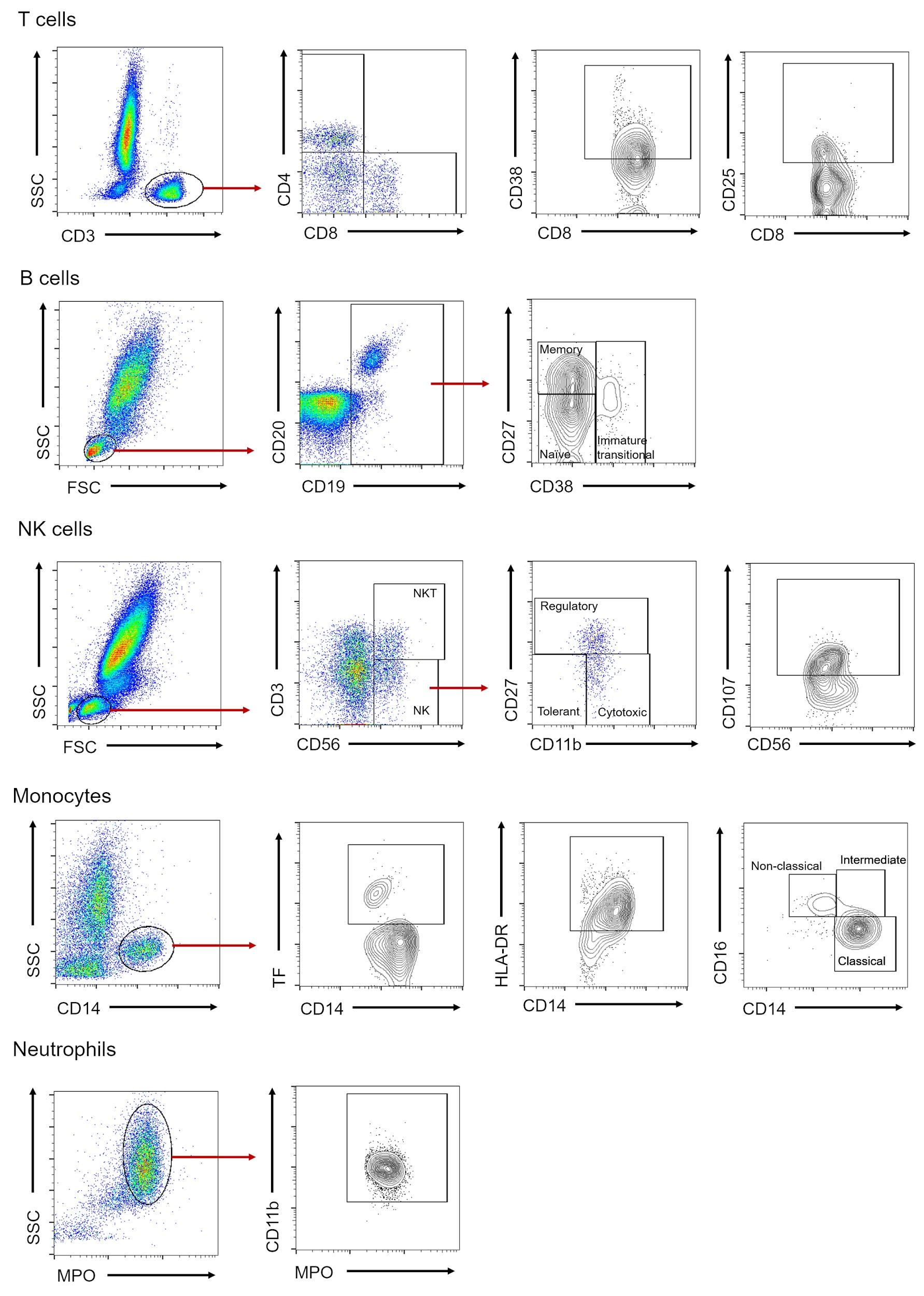
**

**Supplementary Figure 5** – **Gate strategy for the immune profiling of severe COVID-19 patients. (A)** T cells were gated based on their characteristic forward and side scatters and CD3 positivity. T cells were phenotyped according to CD4 and CD8 expression. **(B)** B cells were gated by their characteristic forward and side scatters and the expression of CD19 and/or CD20, and then classified as naïve, immature/transitional or memory phenotypes based on the CD38 and CD27 expression patterns. **(C)** NK and NKT cells were identified by their characteristic forward and side scatters and the expression of CD56 only or CD56 plus CD3, respectively. NK cells were classified as tolerant, regulatory, or cytotoxic phenotypes according to the CD11b and CD27 expression patterns. Cytotoxic activity was confirmed by CD107 surface expression. **(D)** Monocytes were identified according to their characteristic forward and side scatters and CD14 expression, and then classified as classical, intermediate or nonclassical phenotypes according to the CD14 and CD16 expression patters. TF and HLA-DR expression were evaluated. **(E)** Neutrophils were identified by their characteristic forward and side scatters and MPO positivity. CD11b expression was also evaluated.

**Supplementary Table 1** – **Demographic clinical and laboratorial aspects of the patients.**

| **Characteristics outcome^a^** | **Discharged**  **(n=10)** | **Deceased (n=15)** | ***p^c^*** |
| --- | --- | --- | --- |
| Age, years | 57 (42.25 – 63.25) | 56 (51 – 70) | 0.4862 |
| Sex, male | 2 (20%) | 8 (53.33%) | 0.2107 |
| SAPS3 | 63 (56 – 67) | 68 (59 – 78.5) | 0.1903 |
| PaO_2_/FiO_2_ ratio | 160.5 (114.1 - 208) | 130 (80 – 176) | 0.1776 |
| Vasopressor | 5 (50%) | 9 (60%) | 0.6968 |
| Time from symptom onset to blood sample, days | 12 (8 – 17) | 13 (8 – 18) | 0.7359 |
| **Comorbidities** |  |  |  |
| Obesity | 2 (20%) | 2 (13.33%) | >0.9999 |
| Hypertension | 5 (50%) | 8 (53.33%) | >0.9999 |
| Diabetes | 2 (20%) | 5 (33.33%) | 0.6592 |
| Cancer | 2 (20%) | 1 (6.67%) | 0.5435 |
| Heart disease^b^ | 1 (10%) | 1 (6.67%) | >0.9999 |
| **Presenting symptoms** |  |  |  |
| Cough | 7 (70%) | 11 (73.33%) | >0.9999 |
| Fever | 9 (90%) | 12 (80%) | 0.6265 |
| Dyspnea | 8 (80%) | 13 (86.66%) | >0.9999 |
| Headache | 3 (30%) | 2 (13.33%) | 0.3577 |
| Anosmia | 3 (30%) | 4 (26.66%) | >0.9999 |
| **Laboratory findings on admission** |  |  |  |
| White blood cell | 138.5 (91.75 – 149.5) | 168 (123 – 275) | 0.0534 |
| Lymphocyte count, cells/mm^3^ | 1238 (939 – 1527) | 1136 (417 – 1765) | 0.8609 |
| Platelet count, x1000/mm^3^ | 198 (137.5 – 336.5) | 193 (131 – 240) | 0.4373 |
| C Reactive Protein, mg/L | 11.98 (6.41 – 23.44) | 20.33 (12.19 - 27.92) | 0.1963 |
| Fibrinogen, mg/dL | 545.3 (332.8 – 591.7) | 545.3 (453.1 – 621.6) | 0.6505 |
| D-dimer, IU/mL | 3835 (2290 – 11410) | 7401 (3373 – 17065) | 0.3537 |
| IL-6, pg/mL | 23 (15.5 – 50.75) | 56 (28 – 119) | 0.0619 |
| Viral load (RNA copies/mL) | 196433 (3.369 – 3954576) | 78911 (2.105 -  280815) | 0.6540 |

^a^ Numerical variables are represented as the median and the interquartile range, and qualitative variables are represented as the number and the percentage.

^b^ Coronary artery disease or congestive heart failure

^c^ Qualitative variables were compared using the two tailed Fisher exact test, and numerical variables using t test for parametric and Mann Whitney test for nonparametric distributions.

**Supplementary Table 2** – **Quality control of SARS-CoV-2 sequences**

| **Patient code** | **Emerging clade** | **GenBank code** | **Q score SARS-CoV-2** | **SARS-CoV-2 reads (mean)** | **SE (±)** | **Coverage (% mean)** | **SE (±)** | **Depth coverage (mean)** | **SE (±)** |
| --- | --- | --- | --- | --- | --- | --- | --- | --- | --- |
| 1 | 20B | SUB9550672 | >30 | 484 051 | 21 986.16 | 1 561 | 0.012 | 100 | 7.672 |
| 2 | 20B | SUB9550672 | >30 | 3 966 | 31.75 | 12 | 7.492 | 82 | 0.120 |
| 3 | 20B | SUB9550672 | >30 | 23 362 | 86.41 | 67 | 1.550 | 97 | 1.082 |
| 4 | 20A | SUB9550672 | >30 | 5 376 | 84.82 | 23 | 7.201 | 82 | 3.492 |
| 5 | 20B | SUB9550672 | >30 | 91 687 | 499.75 | 272 | 0.183 | 100 | 1.647 |
| 6 | 20B | SUB9550672 | >30 | 1 294 397 | 2 751.61 | 2 968 | 0.023 | 100 | 431.237 |
| 7 | 19A | SUB9550672 | >30 | 963 | 7.72 | 4 | 0.430 | 77 | 0.037 |
| 8 | 20B | SUB9550672 | >30 | 25 960 | 199.85 | 77 | 0.971 | 98 | 0.633 |
| 9 | 20B | SUB9550672 | >30 | 14 342 | 65.62 | 43 | 0.014 | 100 | 0.198 |
| 10 | 20B | SUB9550672 | >30 | 17 966 706 | 4 252 024 | 9 669 | 0.023 | 100 | 30.902 |
| 11 | 20B | SUB9550672 | >30 | 4 095 | 62.07 | 13 | 9.425 | 64 | 0.145 |
| 12 | 20B | SUB9550672 | >30 | 950 508 | 234 655.20 | 3 987 | 0.133 | 100 | 32.832 |
| 13 | 20B | SUB9550672 | >30 | 13 085 | 84.19 | 42 | 3.080 | 93 | 1.801 |
| 14 | 20B | SUB9550672 | >30 | 5 544 | 45.64 | 17 | 0.347 | 98 | 0.112 |
| 15 | 20B | SUB9550672 | >30 | 2 587 016 | 12 847.09 | 7 653 | 0.023 | 100 | 37.941 |
| 16 | 19A | SUB9550672 | >30 | 1 577 | 23.35 | 21 | 2.612 | 83 | 7.837 |
| 17 | 20B | SUB9550672 | >30 | 38 487 | 117.56 | 135 | 4.511 | 88 | 8.872 |
| 18 | 20B | SUB9550672 | >30 | 201 370 | 654.36 | 668 | 0 | 100 | 38.121 |
| 19 | 20A | SUB9550672 | >30 | 7 682 | 85.48 | 23 | 0.032 | 99 | 0.254 |
| 20 | 20A | SUB9550672 | >30 | 724 200 | 2 139.85 | 2 143 | 0.014 | 100 | 6.556 |
| 21 | 20B | SUB9550672 | >30 | 8 220 | 51.03 | 25 | 0.126 | 99 | 0.139 |
| 22 | 19A | SUB9550672 | >30 | 4 098 | 36.52 | 13 | 0.181 | 98 | 0.102 |
| 23 | 19A | SUB9550672 | >30 | 6 363 | 61.78 | 19 | 0.088 | 99 | 0.196 |
| 24 | 20B | SUB9550672 | >30 | 55 489 | 139.73 | 165 | 0 | 100 | 0.415 |
| 25 | 20B | SUB9550672 | >30 | 4 767 728 | 221 803.10 | 28 208 | 9.642 | 67 | 3.969 |

**Supplementary Table 3** – **Quality control of the HERV-K sequences**

| **Patient code** | **Biosample code** | **Q score HERV-K** | **HERV-K reads (mean)** | **SE (±)** | **Coverage (% mean)** | **SE (±)** | **Depth coverage (mean)** | **SE (±)** |
| --- | --- | --- | --- | --- | --- | --- | --- | --- |
| 1 | SAMN19068909 | >30 | 2 493.75 | 67.50 | 97.9 | 0.11 | 22.6 | 0.582 |
| 2 | SAMN19068910 | >30 | 7 322 | 207.90 | 64.95 | 1.84 | 65.05 | 1.835 |
| 3 | SAMN19068911 | >30 | 591.5 | 36.42 | 28.725 | 5.75 | 38.75 | 8.874 |
| 4 | SAMN19068912 | >30 | 6 039 | 152.85 | 100 | 0 | 53.575 | 1.348 |
| 5 | SAMN19068913 | >30 | 1 101.75 | 38.92 | 35.75 | 7.29 | 60.525 | 14.087 |
| 6 | SAMN19068914 | >30 | 5 854.25 | 112.90 | 100 | 0 | 51.925 | 1.006 |
| 7 | SAMN19068915 | >30 | 4 492 | 111.86 | 85.9 | 6.73 | 53.45 | 7.629 |
| 8 | SAMN19068916 | >30 | 5 357.75 | 169.76 | 99.95 | 0.02 | 47.55 | 1.494 |
| 9 | SAMN19068917 | >30 | 1 472 | 55.85 | 64.2 | 1.87 | 20.625 | 1.068 |
| 10 | SAMN19068918 | >30 | 4 938.5 | 113.46 | 100 | 0 | 43.8 | 1.006 |
| 11 | SAMN19068919 | >30 | 2 820.5 | 43.41 | 94.075 | 0.65 | 26.55 | 0.236 |
| 12 | SAMN19068920 | >30 | 1 234.75 | 30.80 | 89.825 | 0.81 | 12.175 | 0.275 |
| 13 | SAMN19068921 | >30 | 1 031.25 | 39.88 | 67.175 | 2.11 | 13.6 | 0.320 |
| 14 | SAMN19068922 | >30 | 841 | 29.15 | 69.65 | 2.66 | 10.75 | 0.185 |
| 15 | SAMN19068923 | >30 | 862.5 | 17.43 | 81.65 | 0.79 | 9.4 | 0.167 |
| 16 | SAMN19068924 | >30 | 1 091.75 | 58.15 | 45.65 | 5.765 | 38.525 | 11.630 |
| 17 | SAMN19068925 | >30 | 1 363 | 38.33 | 87.9 | 0.657 | 13.8 | 0.437 |
| 18 | SAMN19068926 | >30 | 8 974.53 | 199.80 | 100 | 0 | 79.575 | 1.773 |
| 19 | SAMN19068927 | >30 | 24 | 3.5 | 7.4 | 3.4 | 3.8 | 1.13 |
| 20 | SAMN19068928 | >30 | 410.50 | 17.20 | 44.85 | 5.735 | 13.975 | 3.921 |
| 21 | SAMN19068929 | >30 | 2 591.55 | 180.94 | 88.225 | 4.916 | 25.8 | 0.576 |
| 22 | SAMN19068930 | >30 | 562.25 | 29.89 | 8 | 0.671 | 64.675 | 2.686 |
| 23 | SAMN19068931 | >30 | 950 | 50.32 | 63.6 | 1.988 | 12.975 | 0.384 |
| 24 | SAMN19068932 | >30 | 2 830.25 | 74.29 | 99.175 | 0.092 | 20.2 | 3.32 |
| 25 | SAMN19068933 | >30 | 165.51 | 5.88 | 16.525 | 5.394 | 25.3 | 0.649 |
| MC01 | SAMN19070690 | >30 | 4 728.25 | 104.44 | 99.825 | 0.012 | 42.025 | 0.933 |
| MC02 | SAMN19070691 | >30 | 2 718.50 | 50.26 | 98.575 | 0.100 | 24.45 | 0.462 |
| MC03 | SAMN19070692 | >30 | 1 662.53 | 37.60 | 92.875 | 0.532 | 15.875 | 0.391 |
| MC04 | SAMN19070693 | >30 | 191.50 | 9.01 | 15.5 | 4.122 | 18.075 | 3.999 |
| MC05 | SAMN19070694 | >30 | 1 756.25 | 31.99 | 92.4 | 0.507 | 16.85 | 0.275 |
